# Time–Frequency Image-Based Overlapping LPI Radar Signal Detection and Recognition with LPI-YOLO

**DOI:** 10.3390/s26144567

**Published:** 2026-07-18

**Authors:** Hongbin Pan, Honglin Zhuo, Weixuan Chen, Weiru Lai, Yanzhou Zhou

**Affiliations:** 1 School of Automation, Guangdong University of Technology, Guangzhou 510006, China; panhongbin1@mails.gdut.edu.cn; 2 School of Intelligent Manufacturing and Electrical Engineering, Guangzhou Institute of Science and Technology, Guangzhou 510540, China; zhuohonglin@gzist.edu.cn (H.Z.); chenweixuan@gzist.edu.cn (W.C.); laiweiru@gzist.edu.cn (W.L.)

**Keywords:** LPI radar, signal recognition, overlapping radar signals, YOLOv8, time–frequency image, object detection

## Abstract

Accurate recognition of overlapping low-probability-of-intercept (LPI) radar signals under low signal-to-noise ratio (SNR) conditions remains a challenging problem in electronic reconnaissance. Existing methods often struggle to separate and identify multiple overlapping components in time–frequency representations. To address this problem, we present an enhanced YOLOv8-based model, named LPI-YOLO, for processing short-time Fourier transform (STFT) spectrograms. The proposed method integrates a Coordinate Attention (CA) mechanism into the C2f backbone to improve directional feature representation along time and frequency axes. A Transformer encoder is introduced in deeper layers to model global contextual relationships among overlapping signal components. In addition, lightweight GSConv-based bottlenecks and a simplified SimSPPF module are adopted to reduce computational complexity in terms of parameters and FLOPs. Experiments on a simulated dataset containing six modulation types show that the proposed method achieves competitive performance under low SNR conditions (down to −12 dB). Compared with YOLOv8n, the proposed model shows improvements on several challenging modulation classes while maintaining lower computational complexity.

## 1. Introduction

In modern battlefield environments, recognizing radar signal modulation types constitutes a critical component of electronic countermeasures. Low-probability-of-intercept (LPI) radar has become the focus of reconnaissance and anti-reconnaissance efforts [1]. Unlike traditional radars, LPI systems utilize complex modulation techniques to reduce the risk of interception. However, this obscures their time–frequency characteristics and increases the difficulty of modulation recognition for non-cooperative radar systems [2]. Furthermore, as battlefield radar emitters multiply, receivers simultaneously intercept multiple LPI radar signals accompanied by low Signal-to-Noise Ratios (SNRs) and colored noise. As a result, the time–frequency features are severely degraded, and the presence of overlapping signals in both the time and frequency domains further complicates modulation recognition [3]. Therefore, accurately identifying overlapping radar signals under such conditions has become crucial.

Currently, the recognition of LPI radar signals is predominantly achieved through automatic modulation classification (AMC), which generally encompasses three paradigms: hypothesis testing-based methods, feature extraction-based machine learning approaches, and deep learning-based techniques. For instance, Addabbo et al. [4] proposed a likelihood ratio test (LRT) that statistically evaluates the likelihood ratio function of intercepted signals against a noise background, making determinations under appropriate thresholds guided by the Bayesian minimum risk criterion. Concurrently, within the realm of feature extraction-based learning, Meng et al. [5] utilized diagonal integral bispectral features derived from high-order cumulants to classify various modulation types, including linear frequency modulation (LFM), binary phase-shift keying (BPSK), frequency shift keying (FSK), and their hybrids. Their approach achieved a recognition rate exceeding 90% across all evaluated signals at an SNR of 5 dB.

Deep learning methods, conversely, typically require models to assimilate extensive prior information to extract representative features for diverse modulation types, frequently utilizing time–frequency representations as input. In 2016, Zhang et al. [6] extracted radar signal features from both the time and time–frequency domains, employing an Elman neural network (ENN) for classification. While this method attained a 94% accuracy across eight signal types at an SNR of −2 dB, it exhibited subpar performance on Frank and P codes. Subsequently, Wan et al. [7] introduced a multi-component LPI radar signal detection method based on visibility graphs. By integrating the time/frequency projection of the smoothed pseudo Wigner-Ville distribution (SPWVD), they achieved over a 90% correct detection probability for FSK/BPSK composite signals at SNRs > −7 dB. More recently, frameworks combining multi-instance multi-label (MIML) learning with deep convolutional neural networks (CNNs) have shown promise. For example, Pan et al. [8] developed a CNN-MIML framework that achieved over 99% accuracy for four overlapping self-interference signals at 10 dB SNR. Similarly, Chen et al. [9] utilized short-time Fourier transform (STFT) spectrograms combined with a joint semantic deep CNN to recognize time–frequency overlapping signals, yielding a recognition rate greater than 95% at SNRs above 0 dB. Furthermore, an earlier MIML-based algorithm by Pan et al. [10] demonstrated the capability to automatically generate instance representations, effectively decomposing overlapping signals into multiple semantic regions. This specific MIML approach serves as a comparative baseline in Section 4 of this study.

YOLO [11] is a supervised learning-based CNN architecture capable of performing rapid object detection and instance segmentation. By predicting bounding boxes, it accurately acquires the spatial location of targets for subsequent processing. This robust multi-object detection capability endows YOLO with unique advantages in the signal processing domain. Zhu et al. [12] proposed a lightweight YOLO-CJ for composite jamming signal detection, achieving a mean average precision (mAP) of over 96% under SNRs > 0 dB. Wu et al. [13] introduced an attention-embedded YOLOv8 algorithm to separate radar signals under interrupted sampling repeater jamming (ISRJ) conditions, thereby enhancing target measurement performance. Furthermore, Xu et al. [14] improved the YOLOv7 algorithm by converting intra-pulse overlapping signals into SPWVD time–frequency images for feature detection. Relying solely on single-signal training, their method achieved a 91% recognition rate for overlapping signals at a challenging low SNR of −4 dB. These studies collectively indicate that the YOLO framework balances localization precision with computational efficiency, offering a viable approach for end-to-end joint optimization.

Despite these advances, existing YOLO-based methods for overlapping signal detection have been evaluated only at SNRs above −4 dB and achieve approximately 91% accuracy under those conditions; their performance at lower SNRs has not been reported. Furthermore, methods relying on single-signal training do not generalize well when the number and type of simultaneously overlapping signals varies. Consequently, achieving high recognition accuracy for a broad range of LPI modulation types at SNRs below −10 dB remains an open problem.

To address these limitations, we introduce LPI-YOLO, a task-oriented enhancement of YOLOv8 for multi-class overlapping LPI radar signal recognition under low-SNR conditions.

The design is motivated by three observations regarding overlapping signal time–frequency representations. First, individual signal components exhibit distinct directional structures, such as the diagonal trajectories of LFM signals or the horizontal patterns of BFSK signals, which are often degraded by noise and mutual interference. To better preserve such directional information, a Coordinate Attention (CA) mechanism [15] is integrated into the C2f bottleneck of the backbone to enhance feature representation along both time and frequency dimensions.

Second, the morphological characteristics of each signal component, including onset, duration, bandwidth, and modulation trajectory, typically span a wide spatial region and cannot be fully captured by local receptive fields alone. To model such long-range dependencies, a Transformer encoder [16] is introduced in deeper backbone layers to capture global contextual relationships across the feature map.

Third, resource-constrained scenarios require balancing detection performance and computational efficiency. Therefore, a lightweight SimSPPF module is adopted to replace the original SPPF, and GSConv-based GSBottleneck blocks [17] are introduced in the neck to reduce computational cost while maintaining effective feature extraction capability.

Overall, these modifications aim to improve directional feature modeling, global context representation, and computational efficiency for overlapping LPI radar signal recognition.

## 2. Signal Model

### 2.1. Overlapping Signal

In complex electromagnetic countermeasure environments, reconnaissance receivers frequently intercept pulsed signals from multiple emitters simultaneously. These signals exhibit profound overlap in both the time and frequency domains, resulting in severe mutual interference. Assuming that *K* independent radar emitter signals are intercepted within a given observation window, the total received composite aliased signal, denoted as x(t), can be formulated as the linear superposition of the individual signal components and ambient noise:

(1) $$x\left(t\right)=\sum_{i=1}^{K}s_{i}\left(t\right)+n\left(t\right)$$

where $s_{i}\left(t\right)$ denotes the signal originating from the *i*-th radar emitter, and n(t) represents Gaussian noise. Tailored to the intrinsic characteristics of LPI radar waveforms, we selected six representative modulation schemes for modeling and provided a detailed categorization of each signal type.

We selected six representative signal types for our modeling: Linear Frequency Modulation (LFM), Non-linear Frequency Modulation (NLFM), Binary Phase Shift Keying (BPSK), Binary Frequency Shift Keying (BFSK), Frank code, and Multiple-Input Multiple-Output (MIMO), with MIMO also serving as ambient interference. The time-domain mathematical formulations for each signal are detailed in the accompanying Table 1.

In these expressions, the rectangular function rect(t/T) equals 1 when |t|≤T/2 and 0 otherwise, defining the signal’s temporal existence within the pulse duration *T*. *A* is the signal amplitude, *T* is the pulse duration, $f_{0}$ is the initial frequency, $f_{c}$ is the carrier frequency, $\phi_{0}$ is the initial phase, and *k* is the frequency modulation slope. $k_{1}$ and $k_{2}$ denote the linear and nonlinear modulation coefficients, respectively. For the BFSK waveform, $f_{1}$ is the first frequency component, Δf is the frequency step size, and c(t) is the coding sequence.

### 2.2. Noise Model

In practical reconnaissance receivers, ambient noise frequently exhibits a non-uniform power spectral density (PSD), manifesting inherently as colored noise. To rigorously evaluate the robustness of our proposed model against such complex background interference, we incorporated both traditional Additive White Gaussian Noise (AWGN) and colored noise into our simulations. Specifically, the colored noise was generated by passing standard AWGN through a Butterworth low-pass filter. The power spectral distribution of the resulting colored noise is strictly governed by the squared magnitude of the filter’s transfer function, as expressed in Equation (2):

(2) $${\left|H\left(j\omega \right)\right|}^{2}=\frac{1}{1+{\left(\omega /\omega_{c}\right)}^{2N}}$$

where the cutoff frequency $\omega_{c}$ and filter order *N* are configured to simulate real-world non-flat channel interference. Dynamically tuning $\omega_{c}$ adjusts the colored noise bandwidth, effectively emulating complex electromagnetic battlefield conditions for overlapping signals.

### 2.3. Time–Frequency Transformation

Relying solely on independent time-domain waveforms or frequency-domain spectra fails to comprehensively capture the complex, time-varying characteristics of LPI signals within the joint time–frequency domain. To leverage the capabilities of deep convolutional neural networks in two-dimensional (2D) image processing, we employed the Short-Time Fourier Transform (STFT) to convert one-dimensional radar echoes into 2D time–frequency distribution images.

Fundamentally, the STFT partitions a continuous signal into short, localized segments and applies a Fourier transform to each isolated period. By introducing a sliding window function to constrain the signal’s temporal scope, this technique effectively extracts precise instantaneous frequency information. The mathematical calculation of the STFT is expressed as Equation (3):

(3) $$\begin{array}{cc} \mathrm{STFT}\{x(t)\}(\tau ,\omega ) & \equiv X(\tau ,\omega )\\ \; & =\int_{-\infty }^{+\infty }x\left(t\right)w\left(t-\tau \right)e^{-j\omega t}dt \end{array}$$

where x(t) is the input signal, w(t) denotes the window function, τ is the position of the window function along the timeline, and X(τ,ω) represents the result of the Fourier transform.

## 3. Methods

### 3.1. LPI-YOLO Object Detection Network

YOLOv8 [11], a classic algorithm in object detection, integrates target localization and classification tasks into a unified single-stage end-to-end architecture. This design reduces computational redundancy while achieving a favourable balance between detection speed and accuracy. Leveraging the real-time performance and accuracy of the YOLO series, an improved network, LPI-YOLO, is proposed.

**Figure 1:**
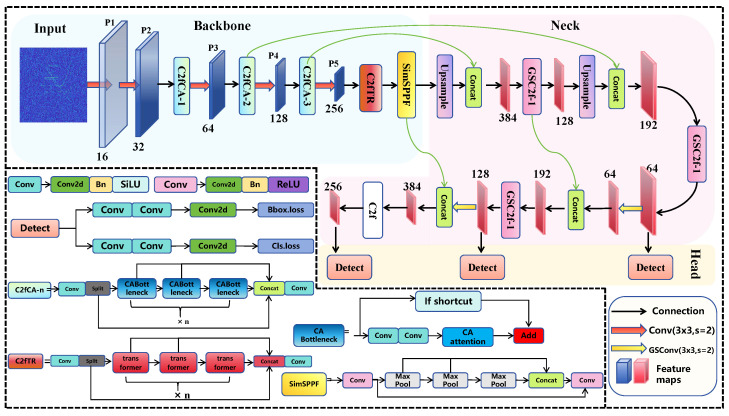
The overall network architecture of the LPI-YOLO.

Overlapping signal time–frequency images present three specific challenges that motivate our architectural choices. First, each modulation type produces a characteristic time–frequency pattern with a well-defined orientation—LFM signals appear as diagonal ridges, BFSK signals as horizontal tone bands, and BPSK signals exhibit phase discontinuities along the time axis. Standard 3×3 convolutions treat all spatial directions symmetrically and cannot exploit this directional structure. We therefore select the Coordinate Attention (CA) mechanism, which applies independent 1D pooling along the horizontal and vertical axes, explicitly encoding the distinct physical meanings of the time and frequency dimensions. Second, overlapping signals often occlude one another partially: the trailing edge of one pulse may be buried within the bandwidth of another. Disentangling such overlap requires reasoning about the global shape of each signal component, which is beyond the reach of purely local convolutions. We address this with a Transformer encoder, whose self-attention mechanism compares every spatial position with every other, allowing the network to associate disconnected fragments of the same signal across the feature map. Third, the C2f module in YOLOv8’s neck contains stacked standard convolutions that contribute disproportionately to the parameter count and FLOPs without commensurate gains in overlapping-signal feature quality. We replace these with GSConv-based GSBottleneck blocks [17], which use depthwise separable convolution to reduce redundancy and channel shuffle to maintain cross-group information flow. The SPPF module is similarly simplified to a Conv-BN-ReLU variant to further reduce computational overhead. The complete architecture is shown in Figure 1.

### 3.2. C2fCA Module

The standard C2f module relies on 3×3 local convolutions, lacking the global long-range dependencies necessary to accurately delineate the Time of Arrival (TOA) and frequency boundaries of overlapping signals. In time–frequency spectrograms, these signals often exhibit highly directional textures—such as the diagonal trajectories of Linear Frequency Modulation (LFM) signals—which are frequently obscured by intense noise. To address this limitation, we propose the C2fCA module by integrating the Coordinate Attention (CA) mechanism [15] into the standard C2f bottleneck. C2fCA preserves the lightweight architecture and gradient flow of the original C2f module while embedding positional information into channel attention. The CA mechanism encodes spatial coordinates along both axes of the time–frequency representation, enabling the module to distinguish signal-related features from background regions more effectively than standard SE attention.

The physical interpretation of the two axes in a radar time–frequency representation motivates the choice of coordinate attention over conventional channel-only attention. The horizontal (time) axis encodes pulse arrival time and duration, which distinguish signals that occupy the same frequency band but arrive at different instants. The vertical (frequency) axis encodes carrier frequency and bandwidth, which separate signals that overlap in time but occupy distinct spectral regions. Standard SE attention collapses both axes into a single channel descriptor, discarding this axis-specific physical information. In contrast, the CA mechanism preserves the independent encoding of each axis through separate 1D pooling operations along the height and width dimensions. For an LFM signal, whose instantaneous frequency varies linearly with time, the diagonal trajectory in the time–frequency plane produces correlated patterns along both axes; the CA mechanism can attend to this joint variation because its direction-aware feature maps retain the spatial structure that SE attention loses. For a BFSK signal, whose frequency hops between discrete tones while remaining constant within each symbol period, the vertical (frequency) encoding captures the tone separation while the horizontal (time) encoding captures the symbol transitions. This axis-aware design is therefore particularly well-suited to the directional textures that characterize radar modulation signatures in the time-frequency domain.

#### 3.2.1. Coordinate Attention Module

**Figure 2:**
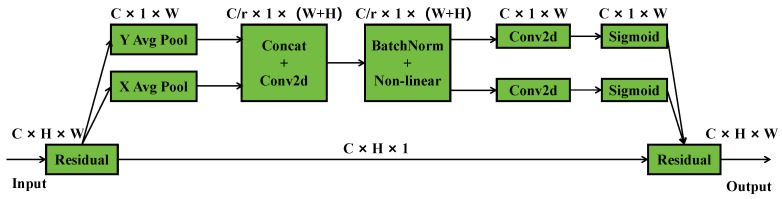
Frame of Coordinate Attention Mechanism.

Unlike traditional Squeeze-and-Excitation (SE) attention, the Coordinate Attention Module (CAM) (illustrated in Figure 2) accounts for the distinct physical dimensions of the time (*X*) and frequency (*Y*) axes in radar spectrograms. Specifically, CAM employs parallel 1D global average pooling to independently aggregate features along both directions. This generates direction-aware feature maps, enabling the network to capture long-range dependencies along one spatial direction while preserving precise positional information along the other for accurate overlapping signal detection.

Assuming the input feature map is denoted as $X\in R^{C\times H\times W}$, the CA mechanism first encodes each channel using pooling kernels of size (H,1) and (1,W) to aggregate features along the vertical and horizontal directions, respectively. Specifically, the output $z_{c}^{h}\left(h\right)$ for the *c*-th channel at height *h* can be expressed as Equation (4). Similarly, the output $z_{c}^{w}\left(w\right)$ for the *c*-th channel at width *w* is expressed as Equation (5).

(4) $$z_{c}^{h}\left(h\right)=\frac{1}{W}\sum_{0\leq i<W}x_{c}\left(h,i\right)$$

(5) $$z_{c}^{w}\left(w\right)=\frac{1}{H}\sum_{0\leq j<H}x_{c}\left(j,w\right)$$

These two transformations aggregate features along two spatial directions. This allows the network to capture long-range dependencies along one direction while preserving precise positional information along the other.

#### 3.2.2. CABottleneck Module

The core component of the C2fCA module is the CABottleneck. Built upon the architecture of the traditional residual block, this structure integrates the Coordinate Attention Module (CAM) to enhance feature representation. For a given input feature $X_{\mathrm{in}}$, the CABottleneck first performs feature transformation via two stacked convolutional layers. Let $F_{\mathrm{conv}1}\left(\cdot \right)$ and $F_{\mathrm{conv}2}\left(\cdot \right)$ denote the sequential CBS (Convolution, Batch Normalization, and SiLU) operations. The intermediate feature map $F_{\mathrm{feat}}$ is computed as Equation (6):

(6) $$F_{\mathrm{feat}}=F_{\mathrm{conv}2}\left(F_{\mathrm{conv}1}\left(X_{\mathrm{in}}\right)\right)$$

In a standard residual block, $F_{\mathrm{feat}}$ is typically added directly to the input via a shortcut connection. However, in the proposed CABottleneck, to capture cross-channel long-range dependencies while preserving precise positional information, $F_{\mathrm{feat}}$ is further processed by the CAM unit, denoted as $f_{\mathrm{CA}}\left(\cdot \right)$. The attention-recalibrated feature map $F_{\mathrm{feat}}^{\prime }$ is computed as follows Equation (7):

(7) $$F_{\mathrm{feat}}^{\prime }=F_{\mathrm{feat}}⊗F_{\mathrm{CA}}\left(F_{\mathrm{feat}}\right)$$

where ⊗ denotes the element-wise multiplication between the original feature map and the attention weights generated by the CAM. Finally, the recalibrated features are aggregated with the input via a residual connection to produce the final output $X_{\mathrm{out}}$, as expressed in Equation (8). This design effectively preserves the original gradient flow while injecting salient positional information:

(8) $$X_{\mathrm{out}}=X_{\mathrm{in}}+F_{\mathrm{feat}}^{\prime }$$

By embedding the CA mechanism within the C2f bottlenecks, the C2fCA module enhances the network’s sensitivity to directional time–frequency patterns.

### 3.3. C2fTR Module

**Figure 3:**
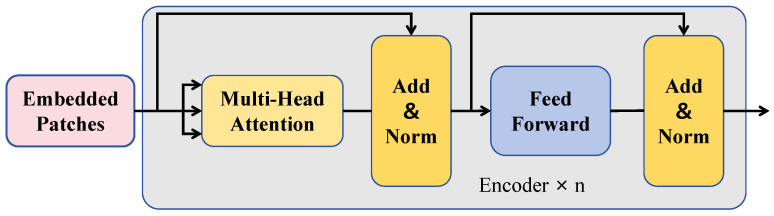
Frame of Transformer Encoder Block.

Preserving the complete geometric morphology of overlapping Low Probability of Intercept (LPI) radar signals is crucial in time–frequency analysis. However, the localized receptive fields of standard Convolutional Neural Networks (CNNs) struggle to capture complete time–frequency trajectories, thereby hindering accurate bounding box regression. To address this, we introduce the C2fTR module, which replaces standard C2f bottlenecks in the deeper backbone layers with Transformer encoder blocks (Figure 3). The self-attention mechanism in the Transformer computes pairwise relations across all spatial positions, providing a global receptive field that complements the local operations of the preceding convolutional layers [16]. The structure of the Transformer encoder block is illustrated in Figure 3.

A signal component in a time–frequency image is defined not by a single local feature but by a coherent trajectory spanning tens to hundreds of pixels across the image. When two signals overlap, a purely local detector observing a small patch may see only a confused superposition and cannot reliably determine whether the observed energy belongs to one signal or another. The Transformer encoder addresses this ambiguity by allowing each spatial position to attend to every other position in the feature map. Through self-attention, a pixel at the intersection of two overlapping signals can reference distant pixels where those signals are well separated—for instance, at their onset or termination regions—and use that global context to resolve the local ambiguity.

Each Transformer module in the main processing path consists of a Multi-Head Self-Attention (MHSA) block and a Feed-Forward Network (FFN). The input feature map $X_{\mathrm{in}}$ is processed by C2fTR for global enhancement Equations (9) and (10):

(9) $$X,X_{\mathrm{branch}}=\mathrm{Split}\left(\mathrm{Conv}\left(X_{\mathrm{in}}\right)\right)$$

(10) $$X_{\mathrm{branch}}^{\prime }=X_{\mathrm{branch}}+\mathrm{MHSA}\left(\mathrm{LN}\left(X_{\mathrm{branch}}\right)\right)$$

The features processed by the MHSA are further transformed via the Feed-Forward Network (FFN), which incorporates residual connections and Layer Normalization, as expressed in Equation (11):

(11) $$Y=X_{\mathrm{branch}}^{\prime }+\mathrm{MLP}\left(\mathrm{LN}\left(X_{\mathrm{branch}}^{\prime }\right)\right)$$

Finally, the C2fTR module concatenates the global features *Y* extracted by the Transformer with the local features *X* from the residual branch. A convolution operation is then applied to fuse these features and generate the final output $Y_{\mathrm{out}}$, as shown in Equation (12):

(12) $$Y_{\mathrm{out}}=\mathrm{Conv}\left(\mathrm{Concat}\left[X,Y\right]\right)$$

By fusing local convolutional features with global Transformer features, the C2fTR module produces a representation that captures both fine-grained details and the overall spatial extent of each signal component. The ablation results (Section 4.8) suggest that this fusion contributes to more accurate bounding box regression for overlapping signals.

### 3.4. SimSPPF Module

YOLOv8 utilizes the Spatial Pyramid Pooling Fast (SPPF) module to aggregate multi-scale contextual information and expand the receptive field through successive pooling operations. In this study, the structural modification of the SimSPPF module primarily involves restructuring the original CBS (Convolution, Batch Normalization, and SiLU) block into a Conv-BN-ReLU (CBR) architecture. Compared with SiLU, which involves a sigmoid-gated nonlinearity, ReLU employs a simple max(0, x) operation that requires fewer floating-point operations per activation. Prior work [18] reports that a single CBR unit achieves approximately 18% faster inference than an equivalent CBS unit under identical conditions. The SimSPPF module adopts this substitution to reduce computational overhead while maintaining multi-scale feature extraction capability.

### 3.5. Integration of C2f and GSConv (GSC2f)

**Figure 4:**
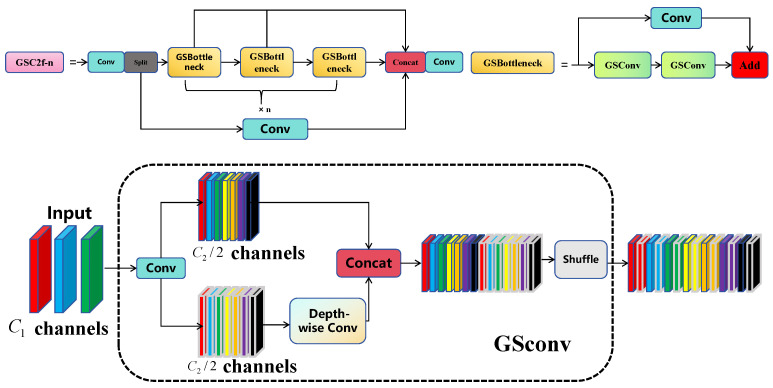
GSC 2f and GSconv Frameworks.

To reduce computational cost, we construct the GSC2f module to reduce the computational redundancy of stacked standard convolutions in YOLOv8’s C2f module. Specifically, standard bottlenecks are replaced with lightweight GSBottlenecks. As shown in Figure 4, GSConv [17] concatenates features from standard and Depthwise Separable Convolutions (DSC) followed by a channel Shuffle operation. In GSC2f, features first undergo 1×1 convolution for dimensionality reduction prior to GSBottleneck extraction. The channel shuffle operation in GSConv enables information exchange across channel groups after depthwise separable convolution, partially recovering the inter-channel communication that standard group-wise convolutions discard. As shown in Section 4, GSC2f reduces the parameter count and FLOPs of the baseline C2f module, thereby lowering the computational cost of the neck network.

## 4. Experiments

### 4.1. Dataset Description

The experiments are conducted on a time–frequency dataset of overlapping Low Probability of Intercept (LPI) radar signals generated using a MATLAB R2024b (MathWorks, Natick, MA, USA)-based simulation framework. Following representative studies in the literature [1,10,14,19], Short-Time Fourier Transform (STFT) is used to convert radar signals into time–frequency spectrograms, and the corresponding simulation parameters are summarized in Table 2.

In the dataset generation process, each signal sample is constructed at a sampling frequency of $f_{s}=600$ MHz with a duration of 0.1 ms. Each spectrogram is formed by superimposing 4 to 6 randomly generated signal components with varying carrier frequencies ($f_{c}$), pulse widths (PW), bandwidths (*B*), and modulation types. The dataset contains 4800 time–frequency spectrograms, covering 12 Signal-to-Noise Ratio (SNR) levels ranging from −12 dB to 10 dB with a step of 2 dB.

To ensure a fair evaluation, 1200 samples are randomly selected from the full dataset and used for training and validation (80%/20% split), while the remaining 3600 samples are reserved for testing. All subsets are generated from the same simulation parameter space and follow identical statistical distributions, while maintaining strict sample-level separation to avoid data leakage.

**Figure 5:**
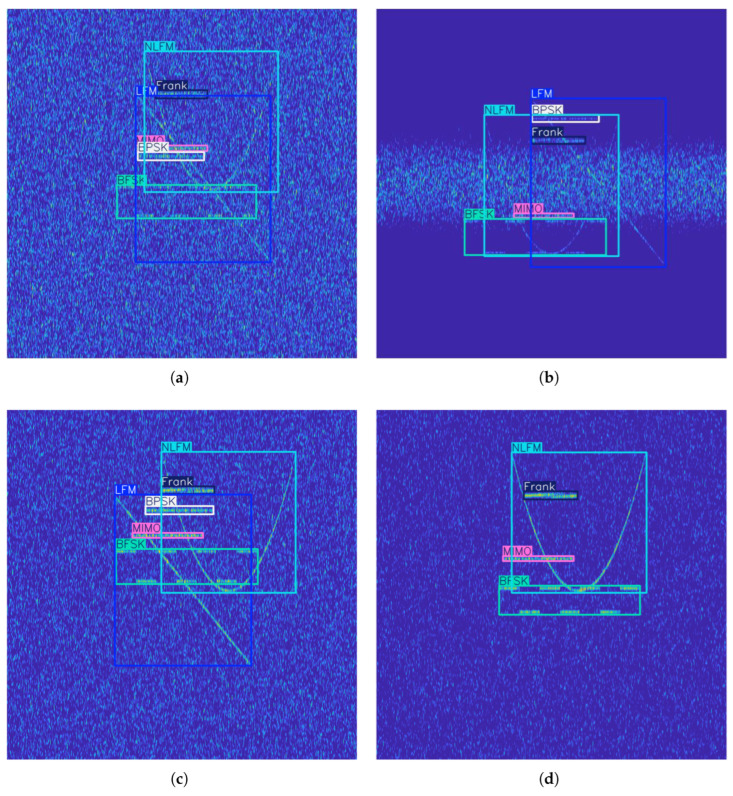
Sample of the dataset annotation section for overlapping signals: (**a**) white noise at −12 dB; (**b**) colored noise at −10 dB; (**c**) white noise at −8 dB; (**d**) colored noise at −6 dB.

For dataset annotation, an adaptive energy-based thresholding method is employed to generate bounding boxes from clean signal spectrograms. These annotations are then transferred to the corresponding noisy spectrograms generated under the same signal parameters, enabling automatic ground-truth labeling. This automatic annotation strategy effectively avoids the potential inaccuracies and inconsistencies caused by manual labeling under low signal-to-noise ratio conditions, ensuring more reliable and reproducible ground-truth generation. Representative annotated spectrograms under different noise types and SNR conditions are shown in Figure 5.

### 4.2. Experimental Configuration

The experiments were conducted on a computing platform equipped with an Intel Core i5-12600K CPU and an NVIDIA RTX 4060 GPU. All detection models were implemented using Python 3.9 and the PyTorch 2.5.0 deep learning framework.

To ensure a fair and consistent comparison among different methods, all models are trained and evaluated under identical experimental settings. The input image resolution is fixed at 640×640 pixels. The batch size is set to 32, and all models are trained for 100 epochs. The Adam optimizer is adopted for all training processes to ensure stable convergence.

The dataset used in this study is generated directly from MATLAB R2024b-based simulations without any additional data augmentation techniques (e.g., flipping, cropping, rotation, or noise injection). All samples are produced strictly according to the signal generation model described in Section 4.1.

During training, all models follow their standard implementation pipelines. All models are evaluated on the same training, validation, and testing splits. Other hyperparameters not explicitly specified follow either the official implementations or default configurations, without model-specific tuning.

The YOLOv8n architecture is used as the baseline framework in this study, upon which the proposed improvements are built.

### 4.3. Analysis of Experimental Results

**Figure 6:**
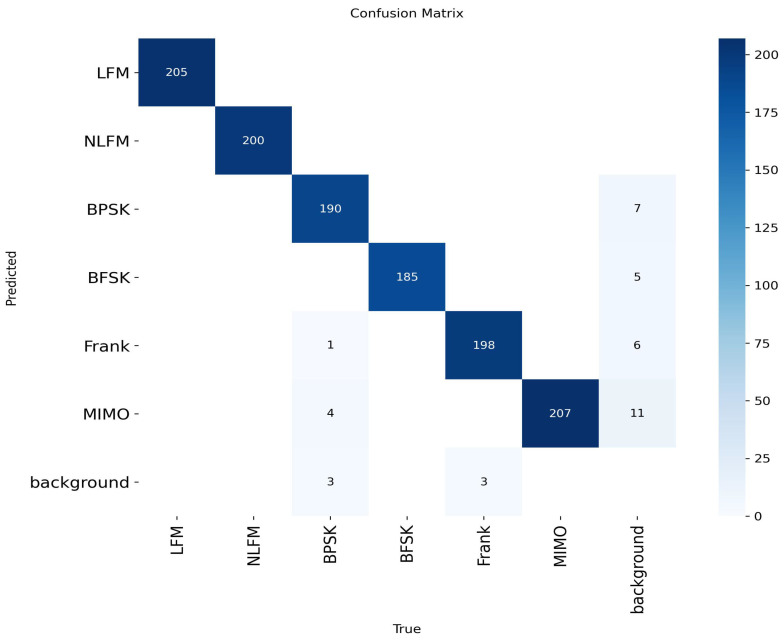
Confusion Matrix.

**Figure 7:**
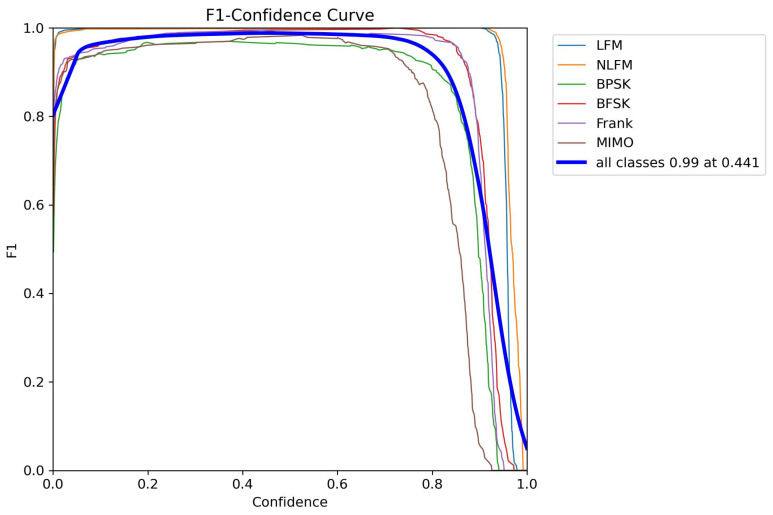
F1 score.

**Figure 8:**
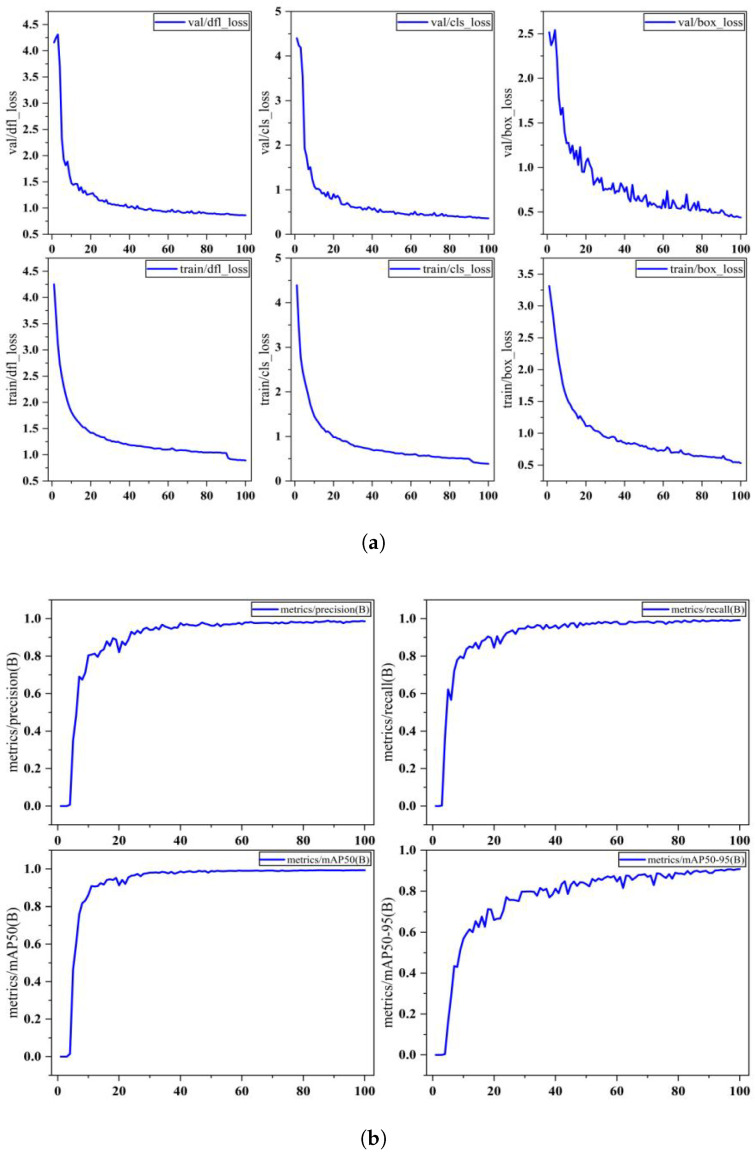
Training loss curves and evaluation metrics: (**a**) training loss curves; (**b**) evaluation metrics.

**Figure 9:**
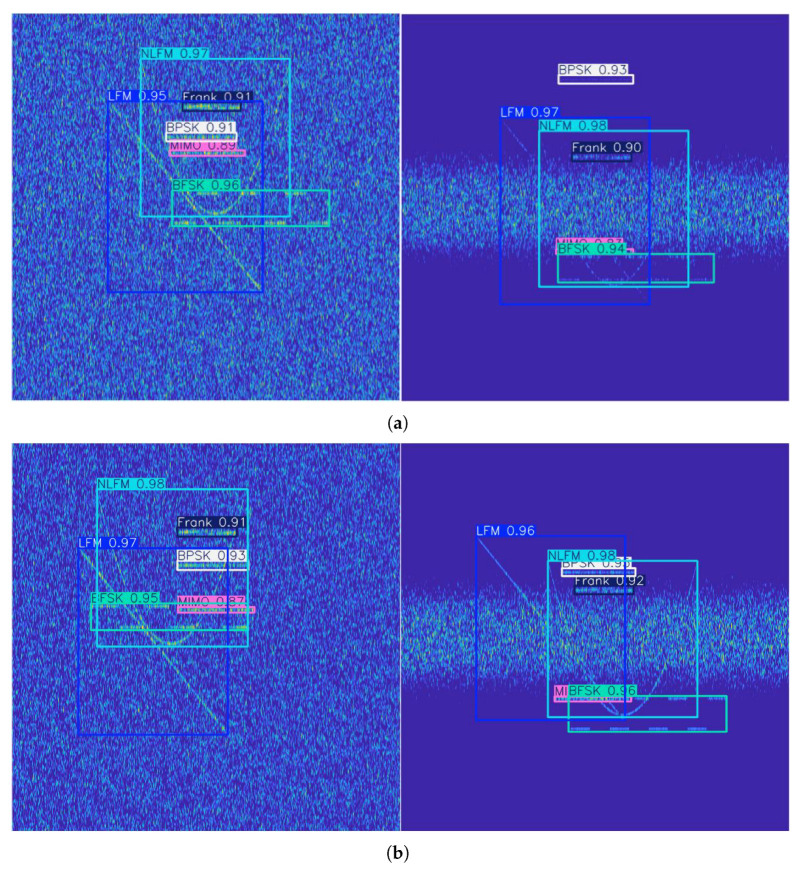
Visualizations of detection results on selected dataset samples. (**a**) Left: −12 dB white noise; right: −10 dB colored noise. (**b**) Left: −8 dB white noise; right: −6 dB white noise.

The improved YOLOv8 network was trained for 100 epochs, with the comprehensive evaluation results presented in Figure 6, Figure 7, Figure 8 and Figure 9. As illustrated by the confusion matrix (Figure 6), predictions are predominantly concentrated along the main diagonal, showing a minimal misclassification rate for overlapping signals. Table 3 details the precision and recall metrics for each class. Correspondingly, the average F1 score (Figure 7) peaks at 0.99 at a confidence threshold of 0.441, indicating a robust balance between precision and recall.

Furthermore, the training loss curves (Figure 8) steadily converge, reflecting consistent improvements in target localization and classification. Both precision and recall exceed 95% after 50 epochs and subsequently maintain stability, highlighting the model’s rapid convergence and adaptability to modulation recognition tasks. Finally, the visualizations of the bounding box detection results are provided in Figure 9.

The training process was monitored through loss curves and evaluation metrics, as shown in Figure 8.

### 4.4. Comparison of Recognition Accuracies

**Figure 10:**
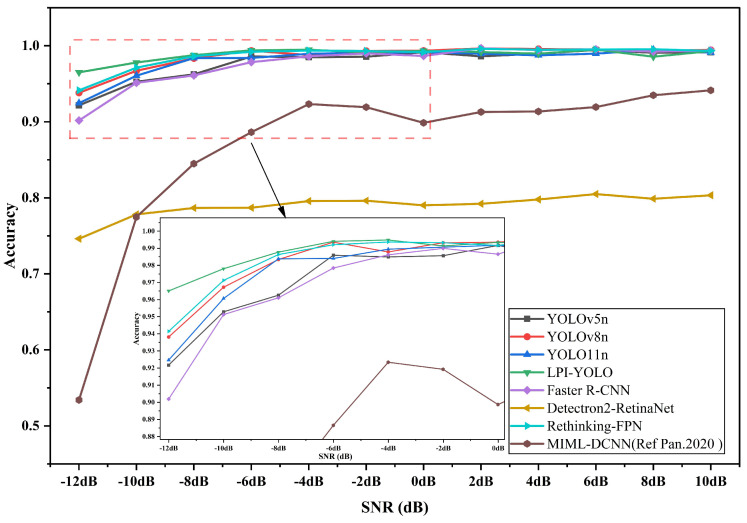
Comparison of recognition accuracy among different algorithms under various SNRs [10].

Comparisons with other algorithms indicate that the proposed LPI-YOLO model achieves competitive performance in recognizing overlapping radar signals, particularly under low SNR conditions. The evaluated object detection methods include YOLOv5n, YOLOv8n, YOLO11n, Faster R-CNN [20], Detectron2-RetinaNet [21], and Rethinking-FPN [22], along with a non-object detection algorithm, MIML [10]. The SNR-accuracy curve in Figure 10 illustrates the final detection results of the LPI-YOLO for signals overlapping under low SNR conditions.

As shown in Figure 10, the proposed LPI-YOLO achieves higher precision than the compared baseline methods across different SNR conditions. In particular, under low-SNR scenarios (from −12 dB to −6 dB), where noise interference and signal overlap present greater challenges for detection, LPI-YOLO maintains competitive performance relative to competing methods.

At an SNR of −12 dB, LPI-YOLO achieves a precision of 96.5%, which is higher than all baseline models, suggesting improved stability under challenging noise-corrupted conditions. In contrast, most comparison methods exhibit more pronounced performance degradation in this region.

As the SNR increases, the performance gap between different methods gradually narrows, and all models converge to relatively high accuracy levels. This suggests that the proposed method is particularly beneficial in low-SNR and heavily overlapping scenarios, where directional feature extraction and global dependency modeling are more important.

Overall, these results indicate the effectiveness of the proposed LPI-YOLO in handling overlapping radar signals under adverse noise conditions, particularly in low-SNR environments.

Table 4 evaluates the computational complexity of LPI-YOLO. Integrating GSBottleneck and SimSPPF reduces the YOLOv8n baseline parameters by 5.65% and GFLOPs by 6.1%. Although LPI-YOLO requires marginally more computation (7.7 GFLOPs, 2.84 M parameters) than YOLO11n and YOLOv5n, its compact size (5.71 MB) offers a competitive trade-off between model complexity and recognition accuracy among the compared methods.

Table 5 summarizes per-class metrics for YOLOv8n and LPI-YOLO under two SNR conditions: averaged over −12 dB to −6 dB, and at −12 dB alone. LPI-YOLO achieves consistent improvements on most categories, with more noticeable gains observed for MIMO and BPSK under low-SNR overlapping conditions.

For MIMO signals, LPI-YOLO increases average precision (from −12 dB to −6 dB) from 0.905 to 0.940 (3.9%) and improves precision at −12 dB from 0.822 to 0.909 (10.6%). For BPSK, the precision at −12 dB increases from 0.896 to 0.945 (5.5%), while the average precision improves from 0.960 to 0.980 (2.1%).

These improvements are attributed to the enhanced feature representation capability of the proposed architecture, where the Coordinate Attention mechanism strengthens directional feature modeling in time–frequency representations, and the Transformer encoder captures long-range dependencies among scattered signal components.

Overall, the results demonstrate that the proposed method provides more stable detection capability in complex overlapping and low-SNR scenarios.

### 4.5. Comparison with Relevant Achievements

Pan et al. (2022) [8] proposed RAUneGAN-MIML, a framework that combines a residual attention-aided U-Net GAN with a multi-instance multi-label (MIML) classifier for overlapping LPI waveform recognition, achieving competitive performance using only single-type signal training. Chen et al. (2023) [9] developed JSL-Net, a joint semantic learning deep CNN that demonstrates strong scalability and robustness to unknown interference when recognizing unseen signal combinations.

**Figure 11:**
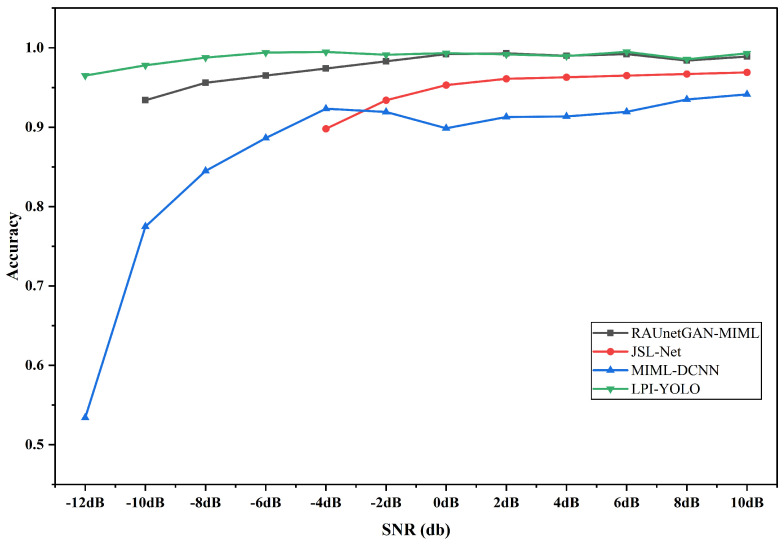
Comparison with related research performance across SNRs.

Figure 11 compares LPI-YOLO with these two methods. The proposed network achieves higher precision than both approaches across all tested SNR levels, indicating its effectiveness for overlapping signal modulation recognition.

### 4.6. Overlap-Density Robustness Evaluation

To further evaluate the influence of overlap density on detection performance, an additional overlap-density robustness experiment is conducted. Three test sets are generated with different numbers of simultaneously superimposed signal components, including 3–4, 4–5, and 5–6 overlapping signals.

The generation procedure of these additional test sets is kept consistent with the dataset generation process described in Section 4.1, including the signal models, parameter sampling ranges, SNR settings, STFT configuration, and automatic annotation procedure. The only difference is the number of signal components superimposed in each spectrogram. These additional datasets are used only for testing and are not included in the training set, validation set, or any fine-tuning process. Therefore, this experiment is designed to evaluate the stability of the trained models under different simulated overlap-density conditions.

As shown in Table 6, the mean precision of all models generally decreases as the number of overlapping signal components increases. This indicates that denser signal superposition introduces stronger time–frequency interference and increases the difficulty of component-level recognition.

LPI-YOLO achieves the highest mean precision from −12 dB to 0 dB under all three overlap-density settings, reaching 0.9324, 0.9253, and 0.9187 for 3–4, 4–5, and 5–6 overlapping signals, respectively. These results suggest that the proposed feature-enhanced structure provides better overall stability as the overlap density increases.

At the extremely low SNR of −12 dB, several baseline models achieve slightly higher precision in individual settings. Specifically, YOLOv5n obtains the highest precision under the 3–4 and 4–5 signal settings, while Rethinking-FPN achieves the best precision under the 5–6 signal setting. Nevertheless, LPI-YOLO remains competitive and consistently achieves higher precision than YOLOv8n across all three overlap-density conditions at −12 dB. This indicates that the proposed method improves the overall robustness to simulated overlap-density variation.

### 4.7. Robustness Evaluation Under Channel Impairments

To comprehensively evaluate the robustness of the proposed method, a further experimental study is conducted under combined channel impairments, including Doppler frequency shift and multipath propagation. Unlike the standard evaluation settings described in previous sections, this experiment considers more realistic and challenging signal propagation conditions, where both frequency offsets and delayed signal replicas are introduced. In addition, additive noise is applied over a wide range of signal-to-noise ratio (SNR) levels from −12 dB to 10 dB.

The trained models are directly evaluated on the constructed test sets without any retraining or fine-tuning. To ensure a fair comparison, all competing methods are tested under identical conditions.

**Figure 12:**
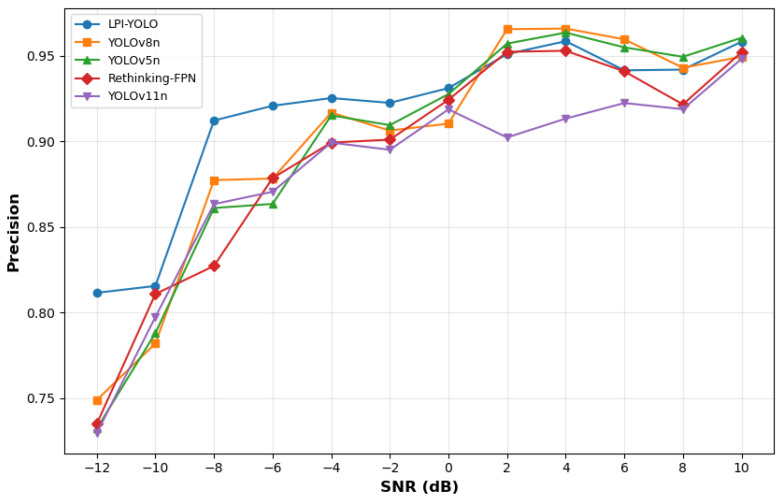
Precision Performance under Doppler and Multipath Channel Impairments.

As illustrated in Figure 12, all models exhibit improved performance as the SNR increases. However, the proposed LPI-YOLO consistently achieves higher precision across most SNR levels, particularly in low-SNR regions (from −12 dB to 0 dB), indicating stronger robustness under channel impairments.

Overall, these results indicate that the proposed method exhibits stable detection performance under combined Doppler and multipath channel impairments.

### 4.8. Ablation Study

Ablation experiments were conducted to evaluate the individual contributions of the proposed modules to the baseline YOLOv8n model, utilizing parameter count (Params), GFLOPs, and recognition accuracy as evaluation metrics.

Integrating the Coordinate Attention (CA) mechanism improved the detection accuracy at −12 dB SNR by 1.4%. The CA mechanism encodes spatial coordinates along both axes of the time–frequency representation, providing direction-aware features that help distinguish signal components from background regions. Incorporating the Transformer encoder further improved the accuracy at −12 dB SNR by 1.2%. The self-attention mechanism provides a global receptive field that complements local convolutions, and the ablation results suggest that this hybrid design benefits the recognition of overlapping signals. Both modules introduced negligible additional computational overhead.

**Figure 13:**
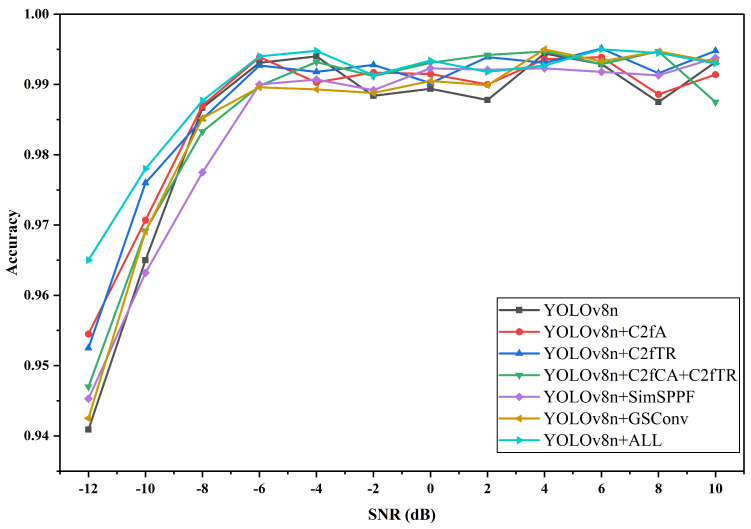
Ablation Experiment Recognition SNR Curve.

Finally, replacing the SPPF module with SimSPPF and introducing GSConv and GSC2f into the neck network decreased the parameter count and GFLOPs by 5.65% and 6.10%, respectively (Figure 13 and Table 7). These comparative results indicate that the CA mechanism and Transformer Encoder primarily enhance detection accuracy, while the GSConv and SimSPPF modules effectively reduce computational costs. Together, these findings indicate the overall effectiveness of the proposed architectural components for recognizing overlapping signals.

### 4.9. Feature Visualization

**Figure 14:**
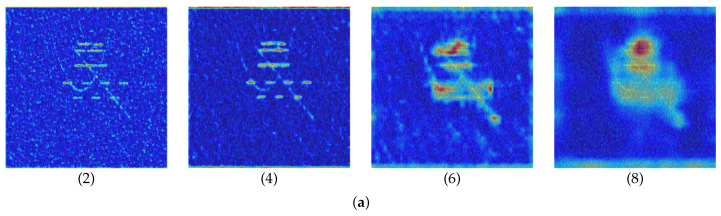

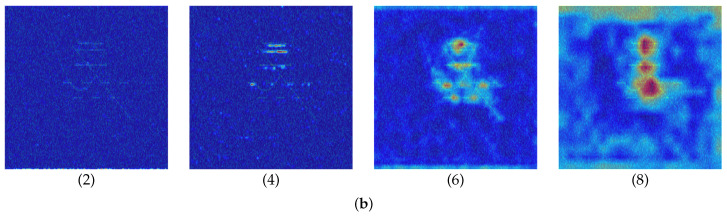
Comparison of Layer 2, 4, 6, 8 Grad-CAM Heatmaps: (**a**) LPI-YOLO Distribution Heatmaps; (**b**) Baseline-YOLOv8n Attention Distribution Heatmaps.

To visually assess the architectural improvements of LPI-YOLO, Gradient-weighted Class Activation Mapping (Grad-CAM) [23] is utilized to generate feature heatmaps. As shown in Figure 14, the Grad-CAM heatmaps indicate that LPI-YOLO concentrates its attention more consistently on the signal-bearing regions compared with the baseline YOLOv8n. Across all four visualized layers, the high-activation areas in LPI-YOLO align more closely with the time–frequency trajectories of the overlapping signal components, whereas the baseline model exhibits more dispersed responses that extend into background regions. These qualitative results suggest that the CA and Transformer modules together guide the network toward more discriminative features for overlapping signal recognition.

## 5. Conclusions

This paper presents LPI-YOLO, an improved object detection network for robust recognition of overlapping Low Probability of Intercept (LPI) radar signals in low-SNR environments. The proposed method transforms radar signals into time–frequency spectrograms using Short-Time Fourier Transform (STFT), and formulates modulation recognition as an object detection task.

To enhance feature representation under challenging conditions, a Coordinate Attention (CA) mechanism is embedded into the C2f backbone to improve directional feature modeling in the time–frequency domain. In addition, a Transformer encoder is introduced to capture global dependencies among overlapping signal components. Lightweight modules, including GSBottleneck and SimSPPF, are further adopted to improve computational efficiency.

Extensive experiments on a simulated dataset show that the proposed method achieves competitive detection performance under various SNR conditions while maintaining a relatively compact model design.

However, the current study is limited to closed-set recognition under simulated conditions, and does not consider unseen modulation types or real-world electromagnetic environments. In addition, hardware-level deployment evaluation and real-time inference benchmarking are not included in this work.

Future work will focus on extending the framework to open-set recognition scenarios, improving generalization to unseen signal distributions, and evaluating the model under real-world and hardware-constrained environments.

## Figures and Tables

**Table 1:** Radar signal model.

Signal	Expression
LFM	$S\left(t\right)=A\cdot \mathrm{rect}\left(\frac{t}{T}\right)\exp\left\{j\cdot \left[2\pi \left(f_{0}t+\frac{kt^{2}}{2}+\varphi_{0}\right)\right]\right\}$
NLFM	$S\left(t\right)=A\cdot \exp\left[j\cdot 2\pi \left(f_{0}t+\frac{1}{2}k_{1}t^{2}+k_{2}t^{3}\right)\right]$
BPSK	$S\left(t\right)=u\left(t\right)\cdot \exp\left[j(2\pi f_{c}t+\varphi_{0})\right]$
BFSK	$S\left(t\right)=A\cdot \exp\left\{j\cdot \left[2\pi f_{1}t+2\pi \Delta fc\left(t\right)+\varphi_{0}\right]\right\}$
Frank	$\begin{array}{cc} S(t) & =A\cdot \mathrm{rect}\left(\frac{t}{T}\right)\exp\left\{j(2\pi f_{c}t+\varphi_{n,k})\right\},\\ \varphi_{n,k} & =\frac{2\pi }{M}\left(n-1\right)\left(k-1\right),\;1\leq n,k\leq M \end{array}$
MIMO	$S\left(t\right)=\sum_{n=1}^{N}A_{n}\exp\left(j(2\pi f_{n}t+\varphi_{n})\right)$

**Table 2:** Signal Parameter Ranges.

Signal	Parameter	Range
LFM	Carrier frequency $f_{c}$	$\left(-1,2.5\right)\times 10^{7}$ Hz
	Pulse width *T*	$\left(1.5,4.5\right)\times 10^{-5}$ s
NLFM	Carrier frequency $f_{c}$	$-1\times 10^{7}$ Hz
	Pulse width *T*	$\left(1.5,4.5\right)\times 10^{-5}$ s
BPSK	Barker code length	[11,13]
	Carrier frequency $f_{c}$	$\left(0,18\right)\times 10^{7}$ Hz
	Pulse width *T*	$\left(3.5,4.5\right)\times 10^{-5}$ s
BFSK	Carrier frequency $f_{c}$	$\left(-6,6\right)\times 10^{7}$ Hz
	Pulse width *T*	$\left(1.5,4.5\right)\times 10^{-5}$ s
Frank	Frequency steps *M*	8
	Carrier frequency $f_{c}$	$\left(15,18\right)\times 10^{7}$ Hz
	Pulse width *T*	$\left(4.0,4.5\right)\times 10^{-5}$ s
MIMO	Carrier frequency $f_{c}$	$\left(3,9\right)\times 10^{7}$ Hz
	Pulse width *T*	$\left(3.5,4.5\right)\times 10^{-5}$ s

**Table 3:** Experimental results.

Class	Images	Instances	Precision	Recall	F1 Score	mAP@0.5
ALL	240	1196	0.986	0.992	0.989	0.993
LFM	205	205	0.997	1.000	0.999	0.995
NLFM	200	200	0.997	1.000	0.999	0.995
BPSK	198	198	0.969	0.957	0.963	0.989
BFSK	185	185	0.990	1.000	0.995	0.995
Frank	201	201	0.989	1.000	0.994	0.995
MIMO	207	207	0.972	0.997	0.984	0.991

**Table 4:** Comparison of computational complexity among different models.

Model	Params (M)	GFLOPs	Size (MB)
LPI-YOLO	2.84	7.7	5.71
YOLO11n	2.58	6.3	5.22
Rethinking-FPN	3.00	8.1	6.04
YOLOv5n	2.51	7.2	5.03
MIML-DCNN	34.97	38.6	387
Detectron2-RetinaNet	36.38	80.47	277
YOLOv8n	3.01	8.2	5.96
Faster R-CNN	41.32	90.74	158

**Table 5:** Per-class detection performance of YOLOv8n and the proposed LPI-YOLO under low-SNR conditions. Metrics are reported as averages over the SNR range of −12 dB to −6 dB (left block) and at −12 dB alone (right block). Bold values in the “All” rows indicate the better result between the two models for each metric.

		−12 dB to −6 dB (avg)				−12 dB			
Model	Class	P	R	mAP@0.5	mAP@0.5–0.95	P	R	mAP@0.5	mAP@0.5–0.95
YOLOv8n	BFSK	0.994	0.993	0.995	0.858	0.986	0.977	0.993	0.830
	BPSK	0.960	0.883	0.964	0.811	0.896	0.758	0.897	0.678
	Frank	0.981	0.988	0.994	0.895	0.970	0.979	0.993	0.853
	LFM	0.998	1.000	0.995	0.954	0.998	1.000	0.995	0.915
	MIMO	0.905	0.977	0.978	0.778	0.822	0.964	0.959	0.701
	NLFM	0.991	0.999	0.995	0.965	0.973	0.996	0.995	0.952
	**All**	0.971	**0.973**	0.987	0.877	0.941	**0.946**	0.972	0.822
LPI-YOLO	BFSK	0.986	0.989	0.995	0.837	0.985	0.961	0.993	0.803
	BPSK	0.980	0.892	0.966	0.813	0.945	0.754	0.899	0.690
	Frank	0.985	0.986	0.995	0.899	0.968	0.971	0.994	0.860
	LFM	1.000	1.000	0.995	0.963	1.000	1.000	0.995	0.917
	MIMO	0.940	0.960	0.982	0.783	0.909	0.926	0.969	0.725
	NLFM	0.994	1.000	0.995	0.973	0.984	1.000	0.995	0.945
	**All**	**0.981**	0.971	**0.988**	**0.878**	**0.965**	0.935	**0.974**	**0.823**

**Table 6:** Precision comparison under different overlap-density settings. Bold values indicate the best result in each column.

Model	Mean Precision (−12 dB to 0 dB)			Precision at −12 dB		
	3–4	4–5	5–6	3–4	4–5	5–6
LPI-YOLO	**0.9324**	**0.9253**	**0.9187**	0.8338	0.8028	0.7840
YOLOv8n	0.9221	0.9167	0.8916	0.8241	0.7837	0.7710
YOLOv5n	0.9037	0.8904	0.8828	**0.8452**	**0.8221**	0.7792
Rethinking-FPN	0.9120	0.8897	0.8829	0.8280	0.7856	**0.7984**
YOLOv11n	0.9019	0.8952	0.8789	0.8281	0.7618	0.7226

**Table 7:** Ablation experimental results at −12 dB SNR. Bold indicates the highest precision.

Model	Params (M)	GFLOPs (G)	Size (MB)	Precision
YOLOv8n	3.01	8.2	5.96	0.941
YOLOv8n + C2fCA	3.1 (+2.99%)	8.4 (+2.44%)	6.17	0.955
YOLOv8n + C2fTR	2.96 (−1.73%)	8.2	5.71	0.953
YOLOv8n + C2fCA + C2fTR	2.97 (−1.33%)	8.3 (+1.22%)	5.92	0.956
YOLOv8n + GSConv	2.79 (−7.31%)	7.4 (−9.76%)	5.58	0.943
YOLOv8n + SimSPPF	3.01	8.1 (−1.22%)	5.96	0.945
YOLOv8n + ALL	2.84 (−5.65%)	7.7 (−6.1%)	5.71	**0.965**

## Data Availability

The data presented in this study are available on reasonable request from the corresponding author.

## Abbreviations

The following abbreviations are used in this manuscript:

LPI	Low Probability of Intercept
YOLO	You Only Look Once
SNR	Signal-to-Noise Ratio
STFT	Short-Time Fourier Transform
LFM	Linear Frequency Modulation
NLFM	Non-linear Frequency Modulation
BPSK	Binary Phase Shift Keying
BFSK	Binary Frequency Shift Keying
MIMO	Multiple-Input Multiple-Output
CAM	Coordinate Attention Module
SPPF	Spatial Pyramid Pooling Fast
GSConv	Group Shuffle Convolution
mAP	Mean Average Precision
Grad-CAM	Gradient-weighted Class Activation Mapping
